# Supplementary material for: Amyloid accelerator polyphosphate fits as the mystery density in α-synuclein fibrils
Source: PLoS Biol. 2024 Oct 31;22(10):e3002650. doi: 10.1371/journal.pbio.3002650 (PMC11527176; doi:10.1371/journal.pbio.3002650)
Supplement: S1 Fig — (A) Density map of patient-derived α-Syn fibrils of the MSA polymorph (PDB: 6XYO), consisting of 2 protofilaments as indicated by the blue and green backbone structures. (B) Density map of patient-derived α-Syn fibrils of the Lewy-fold polymorph (PDB: 8A9L), which consist of a single protofilament. (C) Density map of in vitro-derived α-Syn fibrils (PDB: 6H6B), which consist of 2 protofilaments as indicated by the blue and green backbone structures. A central non-proteinaceous “mystery density” is found in both patient-derived fibrils as indicated by the arrows in 6XYO and 8A9L but is absent in the in vitro-derived α-Syn structure 6H6B. The mystery density is surrounded by residues K43, K45, and His50 in 6XYO and K32, K34, K43, and K45 in 8A9L. The cartoon structures were generated using ChimeraX program. The underlying data can be found in Mendeley (see data statement for details). (DOCX) [file pbio.3002650.s001.docx]

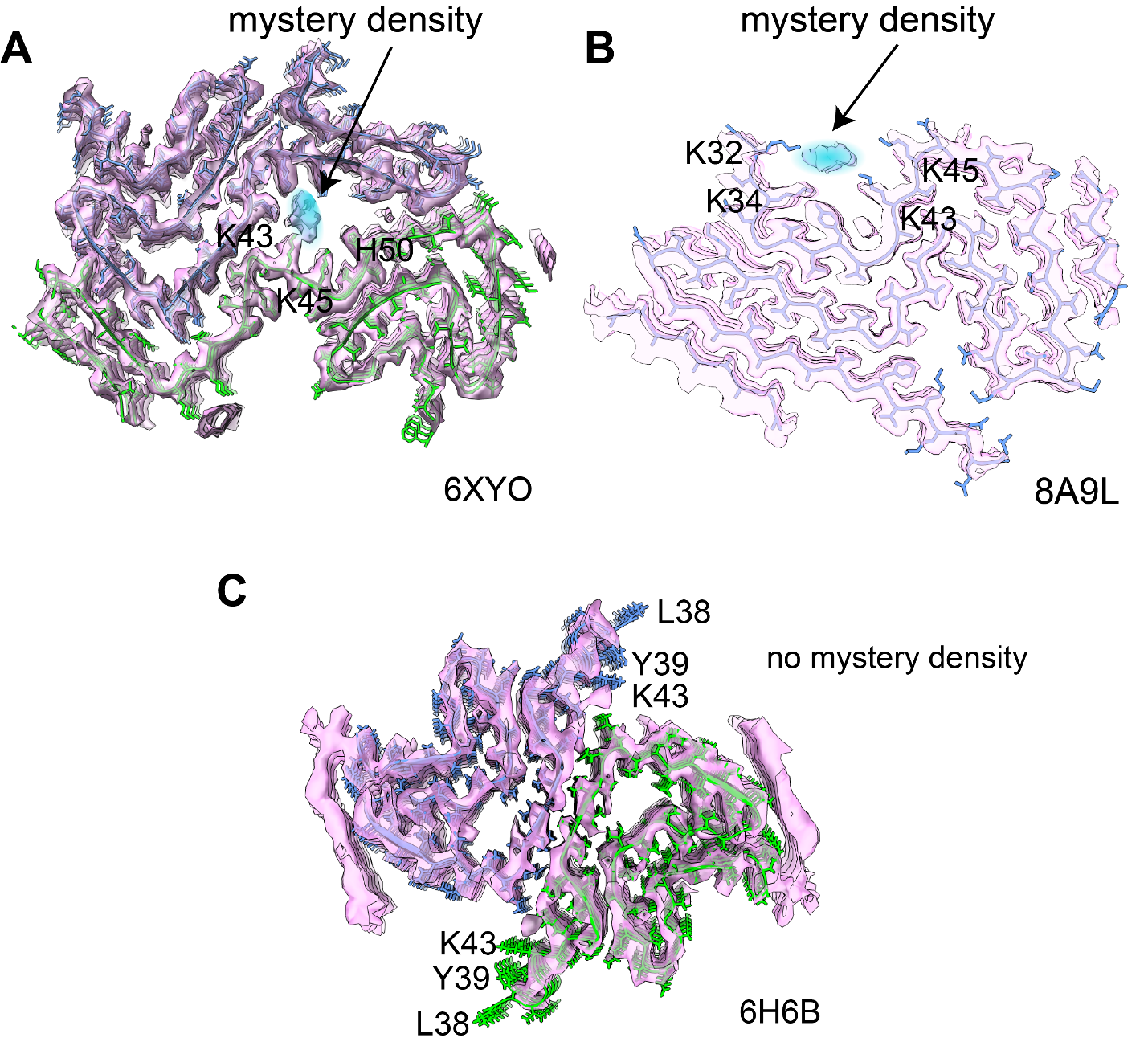


**Figure S1. Cryo-EM structures of α-Syn polymorphs mapped with electron densities.**

**(A)** Density map of patient-derived αSyn fibrils of the MSA polymorph (PDB: 6XYO), consisting of two protofilaments as indicated by the blue and green backbone structures. **(B)** Density map of patient-derived αSyn fibrils of the Lewy-fold polymorph (PDB: 8A9L), which consist of a single protofilament. **(C)** Density map of *in vitro* derived αSyn fibrils (PDB: 6H6B), which consist of two protofilaments as indicated by the blue and green backbone structures. A central non-proteinaceous “mystery density” is found in both patient-derived fibrils as indicated by the arrows in 6XYO and 8A9L but is absent in the *in vitro* derived αSyn structure 6H6B. The mystery density is surrounded by residues K43, K45, and His50 in 6XYO and K32, K34, K43, and K45 in 8A9L. The cartoon structures were generated using ChimeraX program. The underlying data can be found in Mendeley (see data statement for details).

­
