# Supplementary material for: Amyloid accelerator polyphosphate fits as the mystery density in α-synuclein fibrils
Source: PLoS Biol. 2024 Oct 31;22(10):e3002650. doi: 10.1371/journal.pbio.3002650 (PMC11527176; doi:10.1371/journal.pbio.3002650)
Supplement: S2 Fig — (A) PDB structures of an 8A9L monomer, a micelle-bound 1XQ8 monomer, and the α-Syn fibrils 8A9L, 6XYO, and 6H6B. (B) Docked structure of polyP-14 (ball and stick) to α-Syn monomers or fibrils obtained using AutoDock Vina blind docking simulation. PolyP-14 binding residues are labeled and hydrogen bonds are indicated as dashed lines. The cartoon structures were generated using Discovery Studio Visualizer and ChimeraX programs. The underlying data can be found in Mendeley (see data statement for details). (DOCX) [file pbio.3002650.s002.docx]

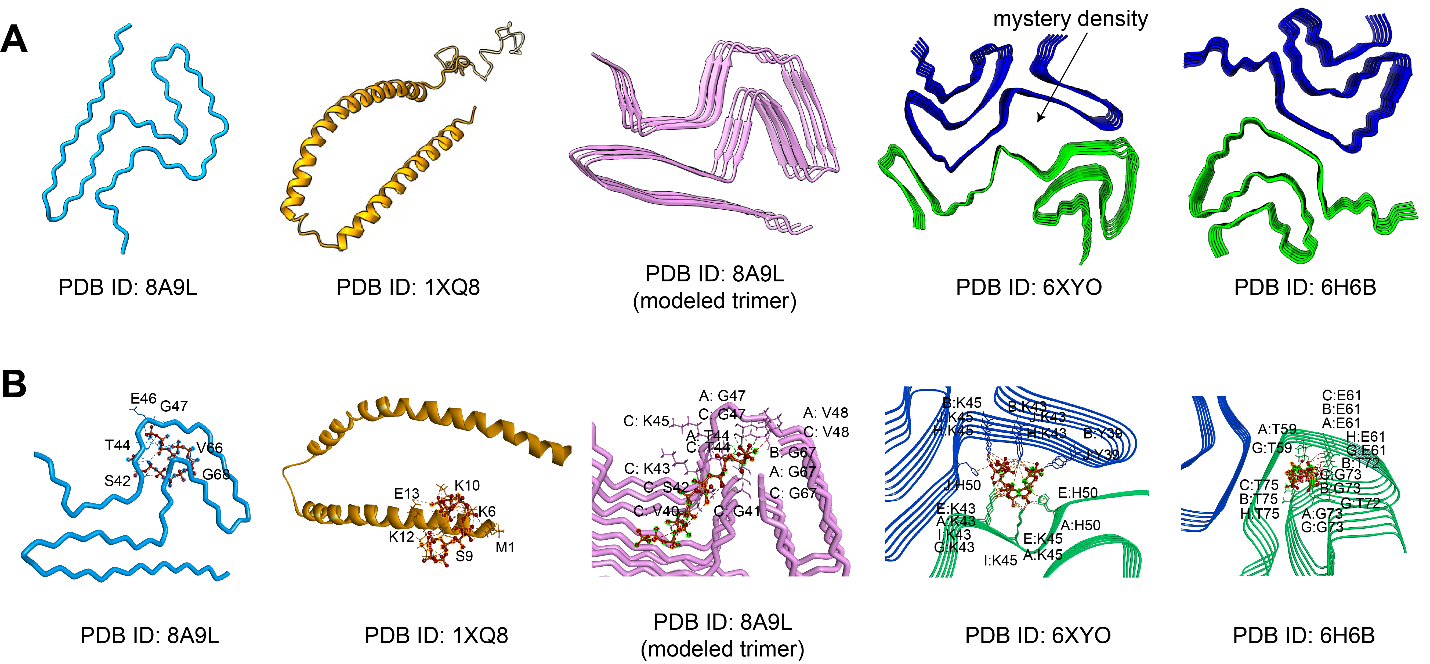


**Figure S2. Structures of α-Syn monomers and selected fibril polymorphs and their molecular interaction with polyP.**

**(A)** PDB structures of an 8A9L monomer, a micelle-bound 1XQ8 monomer, and the α-Syn fibrils 8A9L, 6XYO, and 6H6B. **(B)** Docked structure of polyP-14 (ball and stick) to α-Syn monomers or fibrils obtained using AutoDock Vina blind docking simulation. PolyP-14 binding residues are labeled and hydrogen bonds are indicated as dashed lines. The cartoon structures were generated using Discovery Studio Visualizer and ChimeraX programs. The underlying data can be found in Mendeley (see data statement for details).
