# Supplementary material for: Amyloid accelerator polyphosphate fits as the mystery density in α-synuclein fibrils
Source: PLoS Biol. 2024 Oct 31;22(10):e3002650. doi: 10.1371/journal.pbio.3002650 (PMC11527176; doi:10.1371/journal.pbio.3002650)
Supplement: S3 Fig — (A) A blind docking simulation of polyP-14 (in yellow) matches the unknown electron density in 6XYO. A zoom surface structure prepared using Discovery Studio Visualizer shows the polyP-14 binding pocket fits into the missing non-proteaceous high charge density core. (B) The docked structure of polyP-5 (red) to the Lewy-fold single protofilament structure 8A9L shows its occupancy within the mystery density. The cartoon structure was generated using the ChimeraX program. The underlying data can be found in Mendeley (see data statement for details). (DOCX) [file pbio.3002650.s003.docx]

**
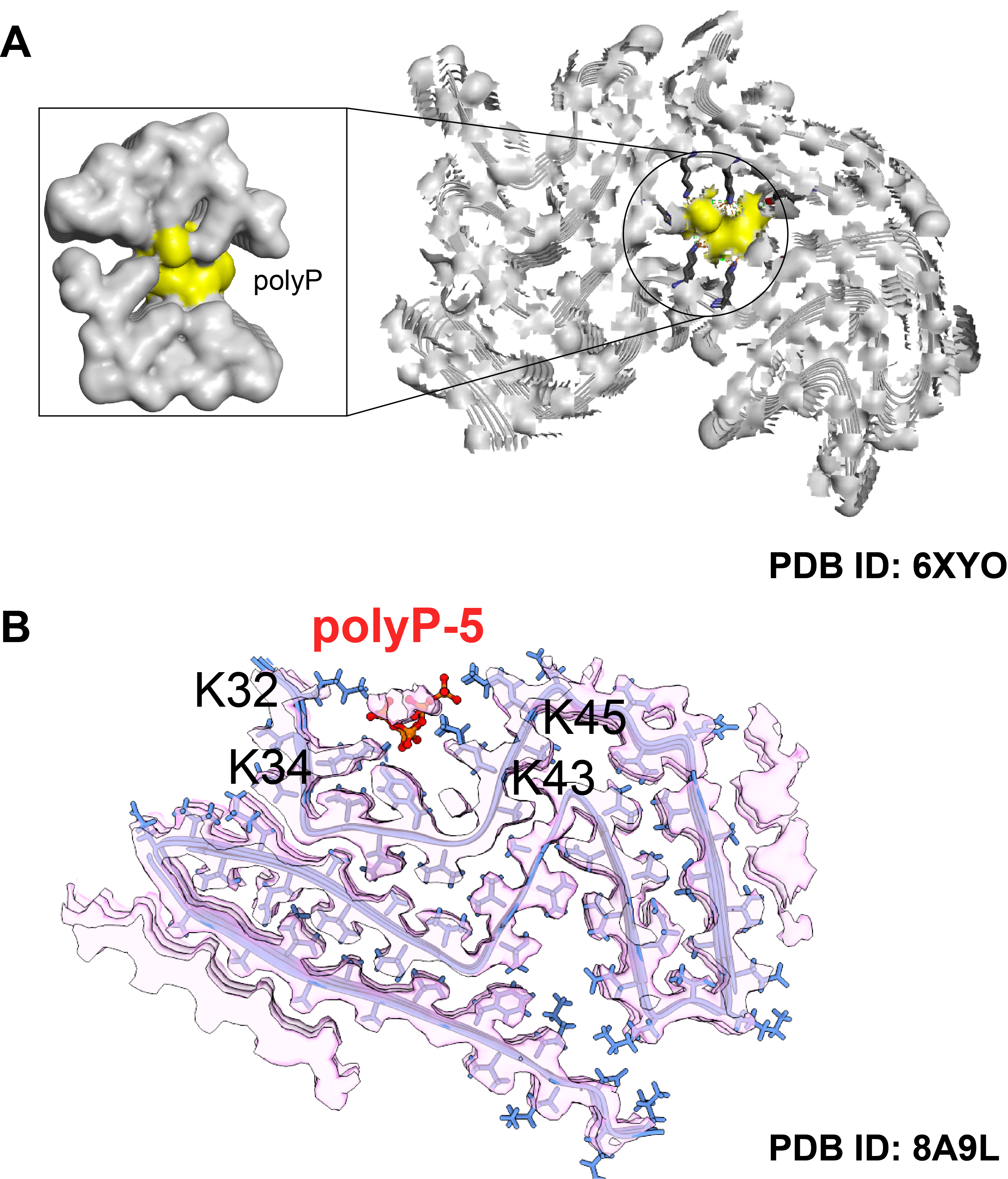
Figure S3. Docking of polyP into the cryo-EM structures of MSA type-I filaments of α-Syn and Lewy-fold single protofilament**. **(A)** A blind docking simulation of polyP-14 (in yellow) matches the unknown electron density in 6XYO. A zoom surface structure prepared using Discovery Studio Visualizer shows the polyP-14 binding pocket fits into the missing non-proteaceous high charge density core**. (B)** The docked structure of polyP-5 (red) to the Lewy-fold single protofilament structure 8A9L shows its occupancy within the mystery density. The cartoon structure was generated using the ChimeraX program. The underlying data can be found in Mendeley (see data statement for details).
