# Supplementary material for: Amyloid accelerator polyphosphate fits as the mystery density in α-synuclein fibrils
Source: PLoS Biol. 2024 Oct 31;22(10):e3002650. doi: 10.1371/journal.pbio.3002650 (PMC11527176; doi:10.1371/journal.pbio.3002650)
Supplement: S4 Fig — (A) Interaction plots between polyP-14 with α-Syn obtained during 100 ns MD simulations using Desmond. The stacked bar plots are normalized over the course of the trajectory where a value of 0.5 suggests that a specific interaction is maintained 50% of the simulation time. A value over 1.0 indicates these α-Syn residues make multiple contacts of the same subtype with the polyP. (B) A timeline representation of α-Syn interaction with polyP-14 over the 100 ns MD simulation. The total contacts between α-Syn and polyP-14 are shown in the top panel. Chain names for individual residues are indicated with a colon. (C) Stability of αSyn-polyP-14 complex measured as a function of protein Cα, and polyP all-atoms root mean square deviation with respect to 100 ns MD simulation using Desmond. (D) Dynamics and solvent accessible properties of polyP-14 complexed with 6XYO obtained from 100 ns MD simulation. The underlying data can be found in Mendeley (see data statement for details). (DOCX) [file pbio.3002650.s004.docx]

**
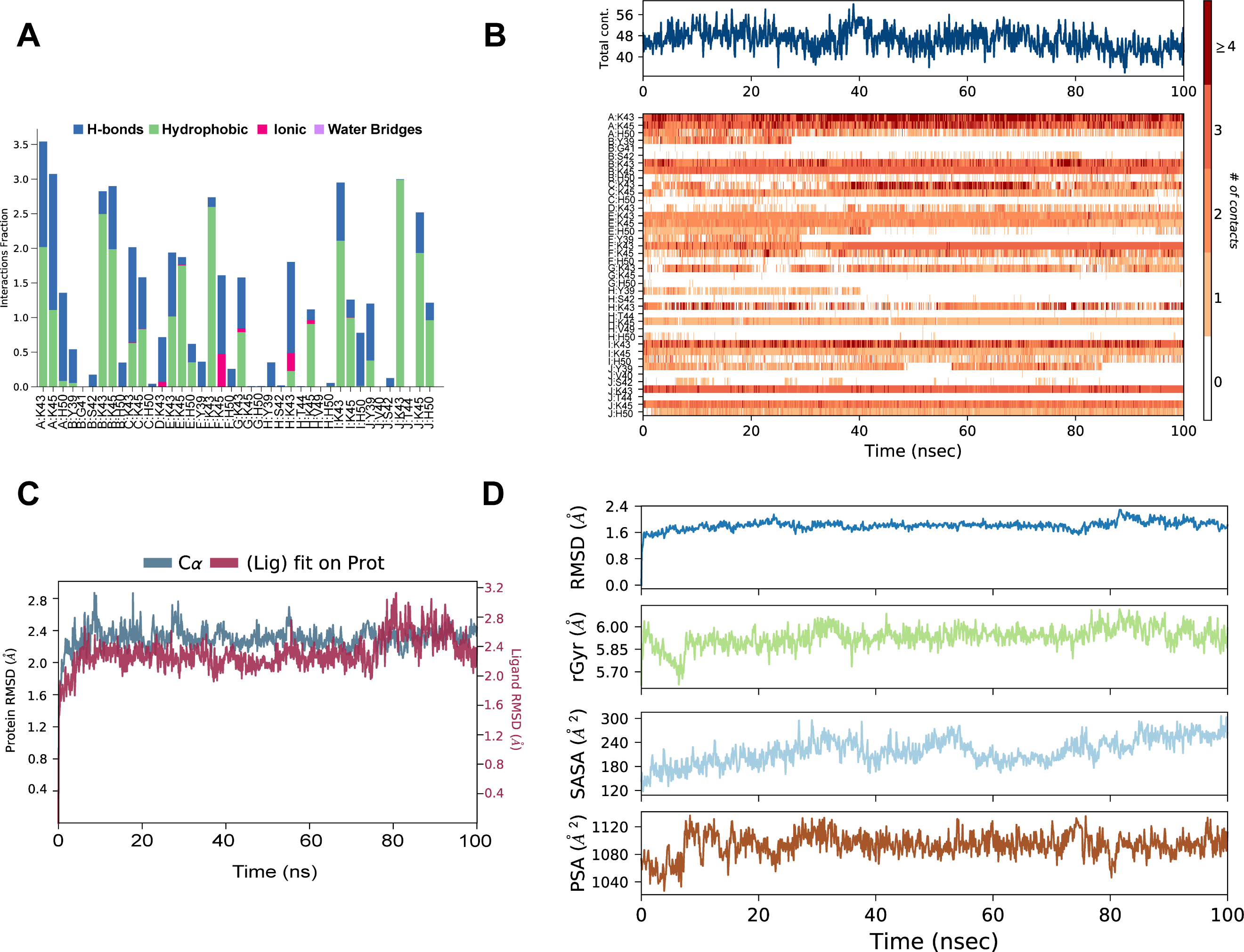
Figure S4. MD analysis assessing the structural and dynamic properties of polyP-14 and α-Syn fibrils.**

**(A)** Interaction plots between polyP-14 and α-Syn (6XYO) obtained during 100 ns MD simulations using Desmond. The stacked bar plots are normalized over the course of the trajectory where a value of 0.5 suggests that a specific interaction is maintained 50% of the simulation time. A value over 1.0 indicates these α-Syn residues make multiple contacts of the same subtype with the polyP. **(B)** A timeline representation of α-Syn interaction with polyP-14 over the 100 ns MD simulation. The total contacts between α-Syn and polyP-14 are shown in the top panel. Chain names for individual residues are indicated with a colon. **(C)** Stability of αSyn-polyP-14 complex measured as a function of protein Cα, and polyP all-atoms root mean square deviation with respect to 100 ns MD simulation using Desmond. **(D)** Dynamics and solvent accessible properties of polyP-14 complexed with 6XYO obtained from 100 ns MD simulation. The underlying data can be found in Mendeley (see data statement for details).

**
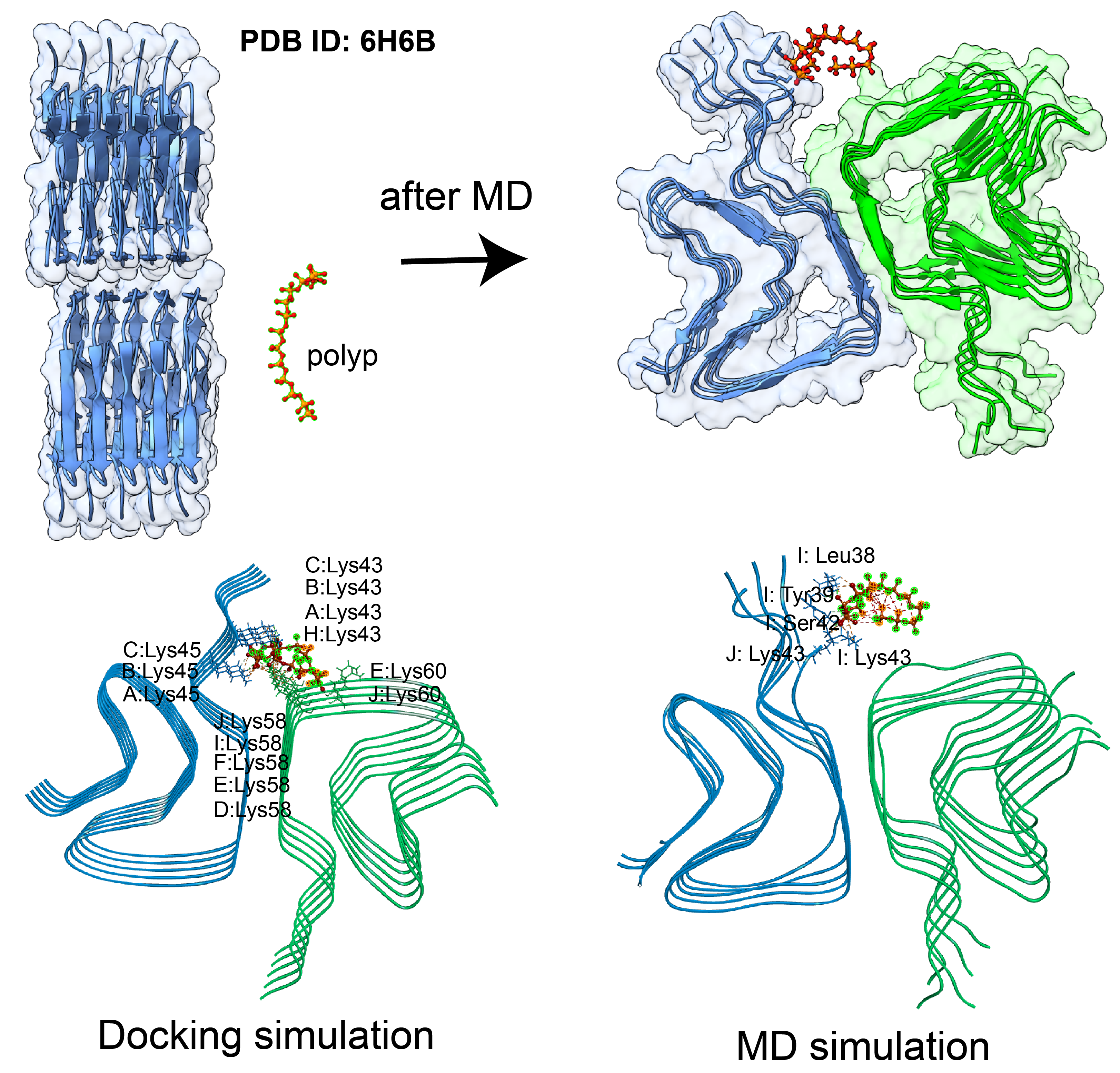
**

**Figure S5. Interaction of polyP-14 and *in vitro* derived α-Syn polymorph** **6H6B**

­­MD snapshots showing the polyP-14 interaction with the cryo-EM structure of *in vitro* derived α-Syn fibrils (PDB ID: 6H6B) before and after 50 ns MD simulation. Cartoon structures shown at the bottom presents the polyP-14 interacting residues in α-Syn 6H6B structure obtained from AutoDock molecular docking simulation (left) with grid center surrounding the binding pocket involving residues K43 and K45, and at 50 ns MD simulation using Gromacs (right). Chain names for individual residues are indicated with a colon.
