# Supplementary material for: Amyloid accelerator polyphosphate fits as the mystery density in α-synuclein fibrils
Source: PLoS Biol. 2024 Oct 31;22(10):e3002650. doi: 10.1371/journal.pbio.3002650 (PMC11527176; doi:10.1371/journal.pbio.3002650)
Supplement: S5 Fig — MD snapshots showing the polyP-14 interaction with the cryo-EM structure of in vitro-derived α-Syn fibrils (PDB ID: 6H6B) before and after 50 ns MD simulation. Cartoon structures shown at the bottom presents the polyP-14 interacting residues in α-Syn 6H6B structure obtained from AutoDock molecular docking simulation (left) with grid center surrounding the binding pocket involving residues K43 and K45, and at 50 ns MD simulation using Gromacs (right). Chain names for individual residues are indicated with a colon. The underlying data can be found in Mendeley (see data statement for details). (DOCX) [file pbio.3002650.s005.docx]

**
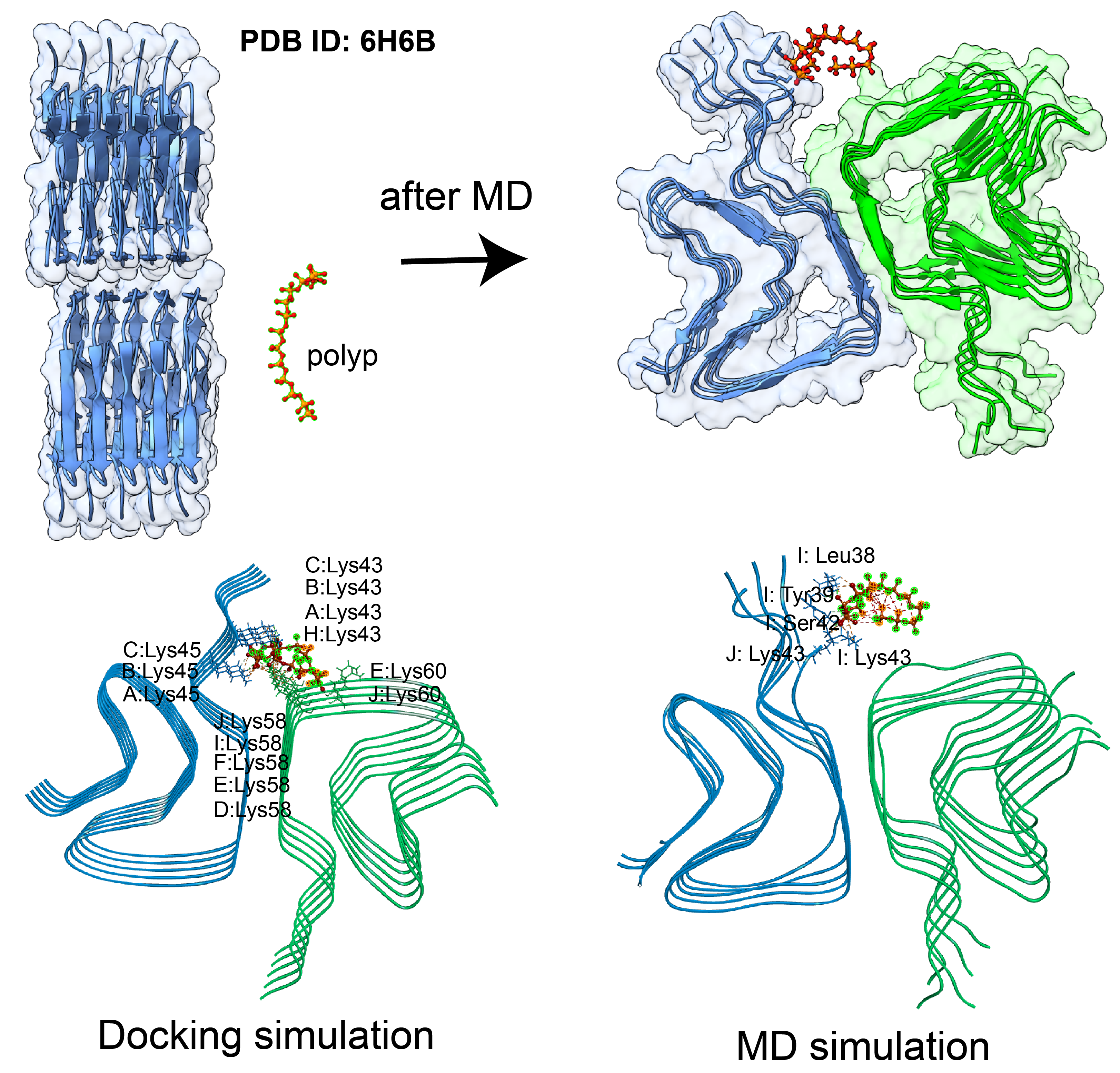
**

**Figure S5. Interaction of polyP-14 and *in vitro* derived α-Syn polymorph** **6H6B**

­­MD snapshots showing the polyP-14 interaction with the cryo-EM structure of *in vitro* derived α-Syn fibrils (PDB ID: 6H6B) before and after 50 ns MD simulation. Cartoon structures shown at the bottom presents the polyP-14 interacting residues in α-Syn 6H6B structure obtained from AutoDock molecular docking simulation (left) with grid center surrounding the binding pocket involving residues K43 and K45, and at 50 ns MD simulation using Gromacs (right). Chain names for individual residues are indicated with a colon.
