# Supplementary material for: Amyloid accelerator polyphosphate fits as the mystery density in α-synuclein fibrils
Source: PLoS Biol. 2024 Oct 31;22(10):e3002650. doi: 10.1371/journal.pbio.3002650 (PMC11527176; doi:10.1371/journal.pbio.3002650)
Supplement: S6 Fig — Arrows point to the α-Syn chain numbers complexed with polyP-14 that exhibit higher fluctuations in the 50–60 residue regions (highlighted in blue) compared to the fibers alone. Residues shaded in gray indicate regions of high RMSF in α-Syn fibers both with and without polyP-14. The underlying data can be found in Mendeley (see data statement for details). (DOCX) [file pbio.3002650.s006.docx]

**
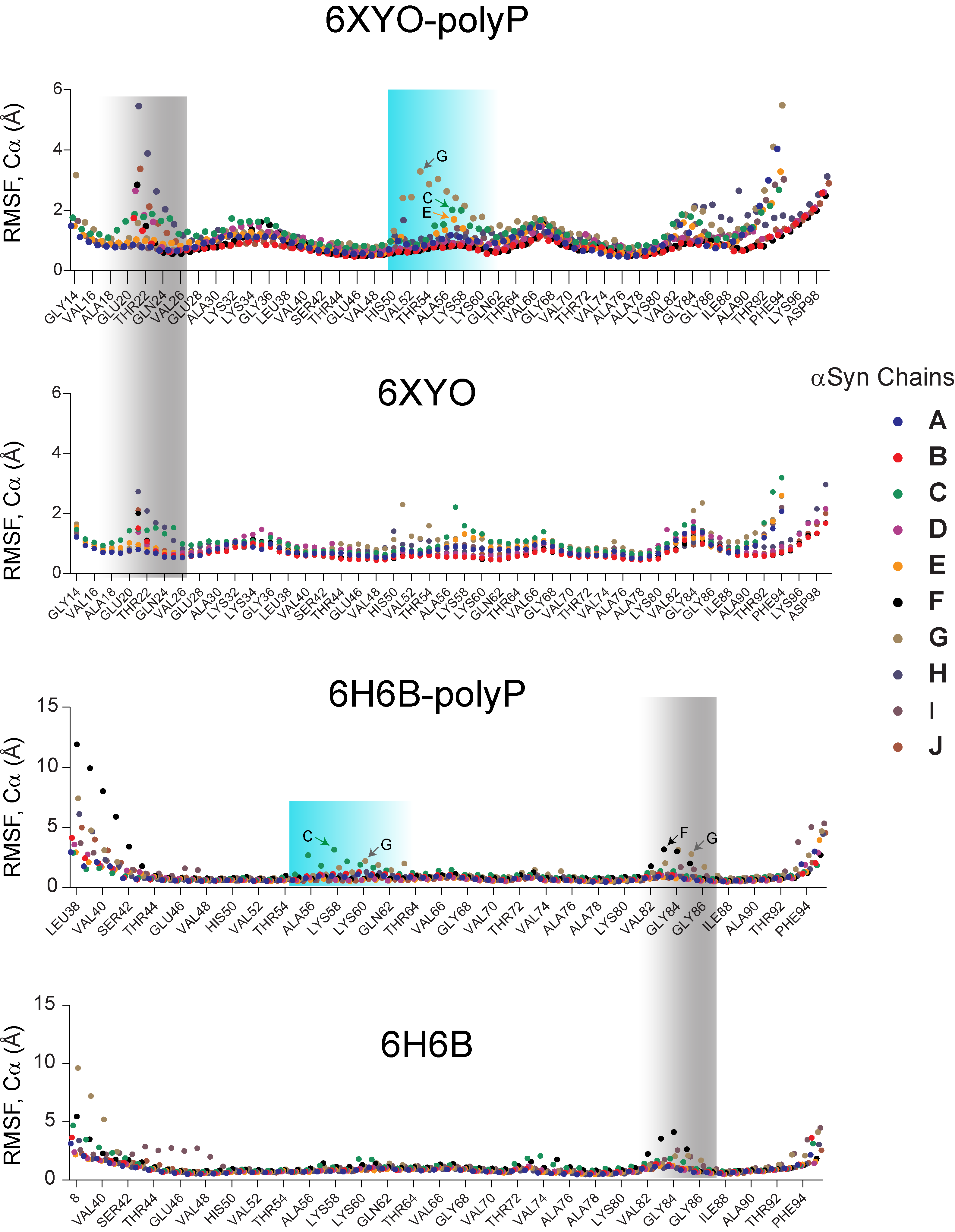
**

**Figure S6.** Root mean square fluctuation (RMSF) plots for individual αSyn molecules in 6XYO and 6H6B, complexed with and without polyP-14 as indicated obtained from 100 ns MD simulation using Desmond. Arrows point to the α-Syn chain numbers complexed with polyP-14 that exhibit higher fluctuations in the 50-60 residue regions (highlighted in blue) compared to the fibers alone. Residues shaded in grey indicate regions of high RMSF in α-Syn fibers both with and without polyP-14. The underlying data can be found in Mendeley (see data statement for details).
