# Supplementary material for: Amyloid accelerator polyphosphate fits as the mystery density in α-synuclein fibrils
Source: PLoS Biol. 2024 Oct 31;22(10):e3002650. doi: 10.1371/journal.pbio.3002650 (PMC11527176; doi:10.1371/journal.pbio.3002650)
Supplement: S7 Fig — Fibrils were prepared in the absence or presence of 500 μM polyP-130 as in Fig 3C. Fibrils were supplemented with SDS to a final concentration of 0.2% (w/v) and pelleted at the indicated time points. The supernatant was loaded onto an SDS PAGE. The total protein was set to 100%; n = 3; mean ± SEM is shown. The underlying data can be found in Mendeley (see data statement for details). (DOCX) [file pbio.3002650.s007.docx]

**Figure S7. Effects of K43, K45 substitution on fibril stability**

Fibrils were prepared in the absence or presence of 500 µM polyP-130 as in Figure 3C. Fibrils were supplemented with SDS to a final concentration of 0.2% (w/v) and pelleted at the indicated time points. The supernatant was loaded onto an SDS PAGE. The total protein was set to 100%; n=3; mean ± SEM is shown. The underlying data can be found in Mendeley (see data statement for details).
