# Supplementary material for: Amyloid accelerator polyphosphate fits as the mystery density in α-synuclein fibrils
Source: PLoS Biol. 2024 Oct 31;22(10):e3002650. doi: 10.1371/journal.pbio.3002650 (PMC11527176; doi:10.1371/journal.pbio.3002650)
Supplement: S1 Table — (DOCX) [file pbio.3002650.s008.docx]

**Table S1** – Primers used for site directed mutagenesis of αSyn

| **Primer** | **Sequence (5’->3’)** |
| --- | --- |
| αSyn K43A forward primer | ctatgtaggctccgcaaccaagg |
| αSyn K43A reverse primer | ccttggttgcggagcctacatag |
| αSyn K45A forward primer | atgtaggctccaaaaccgcggagggagtggtgcatg |
| αSyn K45A reverse primer | catgcaccactccctccgcggttttggagcctacat |
| αSyn K43,45A forward primer | ctatgtaggctccgcaaccgcggagggagtggtg |
| αSyn K43,45A reverse primer | caccactccctccgcggttgcggagcctacatag |
| αSyn H50A forward primer | cactgttgccacaccagccaccactccctccttg |
| αSyn H50A reverse primer | caaggagggagtggtggctggtgtggcaacagtg |
