# Supplementary material for: Amyloid accelerator polyphosphate fits as the mystery density in α-synuclein fibrils
Source: PLoS Biol. 2024 Oct 31;22(10):e3002650. doi: 10.1371/journal.pbio.3002650 (PMC11527176; doi:10.1371/journal.pbio.3002650)
Supplement: Attachment — Submitted filename: Huettemann Response to Reviewers.docx [file pbio.3002650.s009.docx]

**Reviewer #1:** Jakob et al. present a very convincing study of the binding of polyphosphate polymers to alpha-synuclein fibrils both in silico and in vitro with effects in cellulo. They identify potential binding sites on different a-syn fibrils, show that they are compatible with hitherto unassigned "mystery densities" in cryoEM maps and validate this by rational mutagenesis. This sheds striking new light on an important physiologically relevant phenomenon and provides a strong case for the basis of the known binding to fibrils.

We thank the reviewer for their positive remarks and constructive criticism.

I only have minor points to suggest:
1.      Overall provide more information on polyP biology: what is the typical (if any known) molecular weight distribution, how do they form and what is known about their role. Would we expect different polymer lengths to have different effects or is it (as e.g. heparin) largely a question of having a minimum length to promote binding etc. Overall polyanions have remarkable effects in biology - are there any connections to e.g. heparin or is that an unrelated phenomenon as heparin like many other glucosaminoglycans is extracellular?

We have added the following sentence to the introduction: page 3: “*One such modifier is polyphosphate (polyP), a polyanion composed of up to 1000 phospho-anhydride linked phosphates. PolyP is ubiquitously present in every cell and tissue studied, including the brain, where polyP levels are in the micromolar amounts (in Pi-units) and chain lengths are, on average, about 800 residues (17). It remains unclear how these long chain lengths are synthetized and regulated in mammalian cells (17).“*

*Page 13: “These effects become more pronounced as the polyP chain lengths increase, a finding that correlates well with earlier studies, which showed that while polyP chain lengths range widely across mammalian tissues, very long chain (> 800 Pi units) polyPs appear to be the most prominent in mammalian brains (17). Moreover, in contrast to other polyanionic amyloid modifiers such as glucosaminoglycans (i.e., heparin), which are primarily found in the extracellular matrix [40], brain polyP is present both inside and outside of cells as it is released by neurons and taken up by astrocytes* *[41].”*

2.      Fig. S1: It would be helpful to the reader if the two alpha-syn monomers in the structures in panels A and B could be shown in separate colors. Currently it is a bit difficult to make out where the interface between the two protofilaments is. Furthermore, the mystery density could be shown in a different color for ease of visualization.

We thank the reviewer for this suggestion. We have changed the colors for the aSyn monomers accordingly. Please see revised Fig. S1 and the figure legend:

***“Figure S1. Cryo-EM structures of α-Syn polymorphs mapped with electron densities.***

***(A)*** *Density map of patient-derived αSyn* *fibrils* *of the MSA polymorph (PDB:* *6XYO), consisting of two protofilaments as indicated by the blue and green backbone structures.* ***(B)*** *Density map of patient-derived αSyn* *fibrils* *of the* *Lewy-fold polymorph (PDB: 8A9L), which consist of a single protofilament.* ***(C)*** *Density map of in vitro derived αSyn* *fibrils* *(PDB: 6H6B), which consist of two protofilaments as indicated by the blue and green backbone structures. A central non-proteinaceous “mystery density” is found in both patient-derived fibrils as indicated by the arrows in 6XYO and 8A9L but is absent in the in vitro derived αSyn structure 6H6B. The mystery density is surrounded by residues K43, K45, and His50 in 6XYO and K32, K34, K43, and K45 in 8A9L. The cartoon structures were generated using ChimeraX program.”*

3.      Fig. S2: why only use 3 a-syn monomers for 8A9L but 5 for 6XYO and 6H6B?

We used the *αSyn* fibril structures deposited in the PDB database for polyP interaction. The 8A9L structure has one protofilament containing 3 α-Syn monomers while 6XYO and 6H6B structures contain two protofilaments, each containing 5 monomers.
4.      Fig. 1A,B and Fig. S2B: Could the two polyP chains be shown in different colors for ease of visualization? Use same colors in panels A and B and other relevant figures.

We thank the reviewer for the suggestion. We now represent polyP in ball and stick format, colored it by elements (phosphates in orange and oxygen in red) using Discovery Studio Visualizer for ease of visualization.

­­­

5.      Fig. S2B: No mystery density is shown so it is difficult to evaluate that polyP binds to 6H6B's mystery density but not that of 8A9L.

We show the mystery densities in 6XYO and 8A9L in Fig. S1 and explicitly label the 6H6B cryo-EM structure (an *in vitro* fibril structure), which lacks the mystery density.

6.      Figure legend figure 1: H=>G.

Done. Thank you!

7.      "hydrophobic interactions are shown to have minimal contribution in polyP binding to α-Syn fibrils with an average of ~48 total contacts (Fig. S4B)." As far as I can see from panel A, there are plenty of green (hydrophobic) interactions and I simply can't read any hydrophobic contacts into panel B.

Thank you for this comment. We have revised the commented text as “*Interaction analysis shows polyP-14 forms an average of ~48 specific intermolecular contacts with α-Syn fiber structure 6XYO primarily mediated by hydrogen bonding and hydrophobic interactions*”.

8.      "three α-Syn molecules in 6XYO (Chains G, C, E) showed stronger root means square fluctuation in the central region (residues 50-60) in the presence of polyP when compared to 6XYO structure alone." This is definitely intriguing - but could it be an edge/end effect? (i.e. monomer units are less constrained at the ends of fibrils - after all the fibril is not infinitely long but only has 5 units for practical computational reasons).

We thank the reviewer again for this excellent remark. We agree that the monomer units in 6XYO are less constrained at the end of the fibrils. We speculate polyP binding could alter these effects irrespective of their synergistic or antagonistic effect on fibril destabilization. In response to this and reviewer 2’s comment, we have now performed a 100 ns MD simulation for the *in vitro* fibril structure 6H6B. Similar to our observation for 6XYO, polyP binding in 6H6B induces higher RMSF in the region spanning residues ~54-62. The new results are included in Figure S6. Together, these results suggest polyP binding influences a higher RMSF in the region spanning residues ~50-60.

9.      Fig. 4B: x-axis lacks a proper "µM".
Fixed, thank you.
______________________________________________________________________

**Reviewer #2:** In this study, the authors modeled the binding of polyphosphate (polyP) to different alpha-synuclein (aSyn) polymorphs via docking studies and MD simulations. The results suggested that polyP can bind in a lysine-rich pocket previously shown to coordinate an uncharacterized electron-rich density in the structures of aSyn fibrils isolated from the brains of patients. Additional experiments revealed that two lysine residues within the lysine-rich pocket (K43 and K45) are necessary for effects of polyP on aSyn fibrillization, as well as on fibril stability, morphology, and cytotoxicity. The authors conclude that polyP modulates these properties of aSyn by alleviating electrostatic repulsion between lysine residues within the lysine-rich pocket.

This paper is significant in terms of its focus on the structural properties of aSyn amyloid-fibrils, which play a key role in the spread of pathology in the brains of individuals with PD and other synucleinopathy disorders. The demonstration that polyP fits within the mystery electron density observed in cryoEM structures of patient-derived fibrils, and that residues K43 and K45 play a central role in polyP-fibril interactions, represents an important breakthrough that will be of great interest to the PD field.
Strengths of the paper include its comprehensive scope (covering docking and MD simulations, biochemical analyses, and cell culture studies), the rigorous nature of the experimental design, and the high quality of the data. The statistical analyses are appropriate, the methods' section is written in sufficient detail to enable others to replicate the findings, and the supplementary information provides additional useful insights. On the other hand, parts of the manuscript lack details necessary for the reader's understanding, and several errors in the text detract from the overall quality of the paper. The paper would also be improved by adding key points to the discussion and by including additional data and controls. These issues should be addressed in a revised version of the manuscript.

We thank the reviewer for their supportive comments.

p. 4, 'Transmission electron microscopy (TEM) and cryo-EM analysis revealed that polyP-associated alpha-Syn fibrils consist of a single protofilament with a reduced helical twist'; it should be specified that these were recombinant aSyn fibrils, and reference PMID 31533964 should be cited.

We have addressed the comment on page 4: “*Transmission electron microscopy (TEM) [19] and cryo-EM analysis revealed that recombinant α-Syn fibrils formed in the presence of polyP consist of a single protofilament with a reduced helical twist, which has hampered all attempts in obtaining a high resolution cryo-EM structure, leaving the question open as to how polyP interacts with α-Syn fibrils.”*

p. 5, 'These approaches have been successfully used to characterize the interaction of alpha-Syn with small molecules, membranes, metal ions, and other proteins (27, 28)'; this section would be improved by including citations to earlier studies involving MD simulations of the binding of small molecules to aSyn fibrils.
We have added an additional reference.

p. 5, 'as well a'; change to 'as well as a'.
Done.

p. 6, 'the simulations yield in a stronger binding affinity'; delete 'in'.
Done.

p. 6, 'coincides with the strongly electron negative density in 6XYO'; reword (e.g., change to 'strong electron density').
Done.

p. 6, 'each Pi-unit is in contact with 3-4 alpha-Syn molecules from both protofilaments while the free phosphate units at the ends of the polyP-14 structure interact with the first alpha-Syn molecule in each protofilament'; this is an interesting point that isn't evident in the images shown in Fig. 1. It would be helpful to include a panel that better illustrates this point.
We thank the reviewer for this suggestion. We have revised Fig. 1B to show the free phosphate interaction with chains H and G in the 6XYO structure that represents the first a-Syn monomers in each protofilament and separated them from others by using pink dashed lines. We have stated this representation in the figure legend for clarification.

p. 7, 'L-shape grove region'; replace with 'L-shaped groove region'.
Done.

p. 7, 'we found no interactions between polyP and alpha-Syn monomer 8A9L (Fig. 1D)'; for clarity, it would be helpful to indicate whether this part of the analysis involved polyP-5 or -14.
We added polyP-5.

p. 8, 'K45 presented in the D/F alpha-Syn subunits of the one protofilament'; change 'presented' to 'present', and delete 'the' from 'the one protofilament'.
Done.

p. 8, 'Interaction network analysis obtained from 100 ns MD simulation of the alpha-Syn and polyP-14 complex in Desmond revealed that'; it should be specified which fibril structure was examined in this phase of the study.
We have specified the fibril structure “*6XYO complex with polyP-14 used in Desmond MD simulation*” in the revised manuscript.

p. 8, 'MD simulations using the in vitro generated polymorph (6H6B) yielded similar results and, as before, depicted polyP to interact with K43 and K45 residues distributed in one of the protofilaments with additional interactions that involve residues L38, Y38, and S42'; it is unclear why these results differed from those obtained from the docking analysis of 6H6B (i.e., why was polyP binding to the L-shaped groove region not seen here?) Also, the interaction between polyp and K45 is not illustrated in Fig. S5. The figure should be updated accordingly, or the reference to K45 should be deleted from the text. Lastly, 'Y38' should be changed to 'Y39'.
We thank the reviewer for this excellent remark. The blind docking simulation results obtained from AutoDock show that polyP binds to the L-shaped groove with a higher affinity. However, a grid generated around the binding interface involving residues K43 and K45 in AutoDock does not abolish the interaction, rather generated a slightly lower binding energy (a new figure is included in Figure S5). Our motivation for MD simulation was to verify further the in-vacuum docking simulations as molecular interaction studies in an aqueous environment were demonstrated to be an unbiased approach to tracking ligand binding sites in a physiological environment (J. Chem. Inf. Model, 57, 846–863). Our MD results show that polyP does not bind the L-groove, rather binds an interface surrounded by residues K43 and K45. Though the 50 ns MD simulation fails to completely recapitulate the docking results, it correlates to the binding pocket that yields a slightly lower binding energy in AutoDock. We decided to include both the blind and specified grid generated docking results, that explains the necessity of using multiple computational algorithms in addition to experimental validation to identify a ligand binding site in amyloid fibers.

p. 9, 'Based on these results, we now speculate that this increase in root means square fluctuation may indicate an onset of disorder that could destabilize and alter  the molecular packing or both within the β-stacked fibril morphology and might explain why polyp binding to in vitro formed double-twisted fibrils leads to their dissociation into individual protofilaments (19)'; in light of the discussion that follows, this statement would be more convincing if it were shown that the increase in root mean square fluctuations also occurs with the fibril structure of recombinant aSyn, 6H6B'.

We thank the reviewer again for suggesting checking how polyP binding affects 6H6B fiber morphology in MD simulation. We have carried out 100 ns MD simulation for 6H6B in apo and complex with polyP-14. The results agree with our observation for 6XYO, and show that polyP binding induces higher RMSF in the region spanning residues ~54-62. The new results are included in Figure S6.

p. 9, 'a ratio of polyP (in Pi-units) to alpha-Syn monomer of 5:1'; the use of a 5:1 ratio should be justified (e.g., in terms of physiologically relevant aSyn and polyP concentrations). In addition, it would be helpful if the authors included data for mixtures with a lower polyP-aSyn ratio, if these data are available.
We have added the following sentence to the result section: “*We purified the mutant variants and tested their in vitro fibril formation properties using Thioflavin T (ThT) fluorescence in the absence and presence of a 5:1 ratio of polyP to α-Syn. This ratio was found to be optimal in accelerating α-Syn fibrillation in prior in vitro studies^18^, and consistent with reports that the mammalian brain contains between 50 and 100 µM in Pi units [35] and between 5 and up to 50 µM α-Syn [18, 36].”*

p. 11, 'had no significant affect the fibril morphology of the mutant variant'; change to 'had no significant effect on the fibril morphology …'
Done.

p. 12, 'After 5 min of incubation, we monitored the changes in ThT fluorescence as a measure of fibril destabilization and dissociation'; the data are difficult to interpret in light of potential quenching effects of SDS on ThT fluorescence. This question could be addressed by performing fluorescence measurements immediately after adding the SDS, or by using a different method to monitor aSyn fibril levels such as ultracentrifugation coupled with Western blot analysis.
While we appreciate this concern, we do not think that additional experiments would be necessary as any potential quenching effects of SDS on ThT fluorescence would be the same for all samples.

p. 12, 'unaffected by the presence polyP'; change to 'presence of'.
Done.

p. 14, 'PolyP binding to these oligomers serves a similar role by charge-neutralizing the core cluster of positive residues, thus accelerating fibril formation and increasing fibril stability'; this statement might seem confusing in light of the earlier statement that 'an onset of disorder that could destabilize and alter the molecular packing or both within the beta-stacked fibril morphology' (p. 9). This part of the discussion would be improved by clarifying that polyP destabilizes interactions between protofilaments but stabilizes the cross-beta structure within each protofilament.
We have changed this sentence accordingly:

page 15*: “PolyP binding to these oligomers serves a similar role by charge-neutralizing the core cluster of positive residues, thus accelerating fibril formation and increasing the cross-beta structure within each protofilament”.*

p. 15, 'four disease-associated mutations, i.e., E46K, H50Q, G51D, and A53T'; genetic data suggest that H50Q is not a pathogenic variant of aSyn (e.g., see PMID 29398121).

We removed H50Q from the list.
page 15: “*In fact, three disease-associated mutations, i.e., E46K, G51D, and A53T are part of this loop region [43].”*

p. 17, 'fixed number of simulated particles (P), volume (V) and temperature (T) throughout the simulation) and NPT (i.e., a fixed number of simulated particles (P), pressure (P) and temperature (T) throughout the simulation) ensembles'; presumably the 'number of simulated particles' should have the abbreviation 'N'?
Thank you. We have corrected this abbreviation.

p. 21, 'In vivo toxicity assays'; suggest replacing 'in vivo' with 'cell culture'.
Done

Fig. 1G, legend: the letter should be changed from 'H' to 'G', and the identity of the aSyn structure should be indicated.
Done. Thank you!

Fig. 3C, legend: 'Fibrils of WT alpha-Syn (black/grey) or α-Syn [K43A,K45A mutant] (purple)'; replace 'purple' with 'light and dark purple'.
Done. Thank you!

Fig. 4B: the data would be more convincing if additional pairwise comparisons were presented (e.g., WT aSyn at 0 versus 25 micromolar or 0 versus 50 micromolar polyP-130), and for this, a two-way ANOVA could be useful.
We thank the reviewer for this suggestion and have now performed a two-way ANOVA analysis. We revised Fig. 4B accordingly.

Fig. S4 legend: the aSyn structure should be specified, and it should be indicated that the results were obtained using the Desmond method. Also, in panel A, it is uncertain how the data were normalized. It is stated that a value of 0.5 corresponds to a given interaction being maintained 50% of the simulation time, but then it's unclear why certain segments representing different interaction types have lengths >1. This point should be addressed.

The stacked bar charts are normalized over the course of the trajectory: for example, a value of 0.7 suggests that 70% of the simulation time the specific interaction is maintained. Values over 1.0 are possible as some protein residue may make multiple contacts of the same subtype with the ligand. We have clarified this in the figure legend as suggested.
