# Supplementary material for: Amyloid accelerator polyphosphate fits as the mystery density in α-synuclein fibrils
Source: PLoS Biol. 2024 Oct 31;22(10):e3002650. doi: 10.1371/journal.pbio.3002650 (PMC11527176; doi:10.1371/journal.pbio.3002650)
Supplement: Attachment — Submitted filename: Comments to the Reviewers PLOS Bio final.docx [file pbio.3002650.s010.docx]

Dear Richard,

Thank you for the wonderful news. We have addressed all the comments and hope you will find our ms acceptable for publication in PloS Biology.

(A)We would like to suggest the following minor modification to the title:

“Amyloid accelerator polyphosphate is the mystery density observed in structures of α-Synuclein Fibrils and enhances fiber stability"

Thank you for your suggestion. Because we have not absolutely demonstrated that it is polyP in the patient fibrils (a task that is close to impossible), we do not want to make such a definitive statement in the title.

(B) You may be aware of the PLOS Data Policy, which requires that all data be made available without restriction: <http://journals.plos.org/plosbiology/s/data-availability>. For more information, please also see this editorial: <http://dx.doi.org/10.1371/journal.pbio.1001797>

-Deposition in a publicly available repository. Please also provide the accession code or a reviewer link so that we may view your data before publication.

Regardless of the method selected, please ensure that you provide the individual numerical values that underlie the summary data displayed in the following figure panels as they are essential for readers to assess your analysis and to reproduce it:

Figure 2A-B, 3A-C, 4B, S4A-D, S6

We have deposited all the underlying data for Figures 1, 2A-B, 3A-C, 4B, S4A-D, S6, S7 in Mendeley.

(C) Please also ensure that each of the relevant figure legends in your manuscript include information on *WHERE THE UNDERLYING DATA CAN BE FOUND*, and ensure your supplemental data file/s has a legend.
We have added this information to all the figure legends accordingly.

(D) Please ensure that your Data Statement in the submission system accurately describes where your data can be found and is in final format, as it will be published as written there.
We have added this information to the Data Statement.

(E) Per journal policy, if you have generated any custom code during the course of this investigation, please make it available without restrictions. Please ensure that the code is sufficiently well documented and reusable, and that your Data Statement in the Editorial Manager submission system accurately describes where your code can be found.

Please note that we cannot accept sole deposition of code in GitHub, as this could be changed after publication. However, you can archive this version of your publicly available GitHub code to Zenodo. Once you do this, it will generate a DOI number, which you will need to provide in the Data Accessibility Statement (you are welcome to also provide the GitHub access information). See the process for doing this here: <https://docs.github.com/en/repositories/archiving-a-github-repository/referencing-and-citing-conten>

not applicable

______________________________________________________________________
Reviewer #1: The authors have addressed my concerns very well.

Thank you

Reviewer #2: The authors have responded well to the initial comments, and as a result the paper is much improved.

There are a few remaining issues that should be addressed in a revised version of the manuscript:

p. 7, 'L-shaped grove region'; replace 'grove' with 'groove'.
done

Fig. 1B, legend, 'The two free phosphate units interacting with the first two alpha-Syn molecules facing opposite in each protofilament (chains G and H) are shown in pink lines'; replace with 'Shown are interactions (represented by pink lines) representing one free phosphate unit interacting with the first two alpha-Syn molecules facing opposite in each protofilament (chains G and H)'.
done – thank you for this suggestion.

Fig. 1G legend: the identity of the aSyn structure analyzed should be specified. For Fig. S4, 6XYO should be specified earlier in the legend.
done and done – thank you for catching this.

Fig. 4B: the two-way ANOVA should be used to assess whether additional pairwise comparisons across subgroups defined by different polyp-130 concentrations (e.g., WT aSyn at 0 versus 25 micromolar or 0 versus 50 micromolar polyP-130) show significant differences.
We have used 2-way ANOVA for pairwise comparisons across subgroups as suggested – none of those showed significant differences. We added this information to the figure legend.

Response to the question about SDS effects on ThT fluorescence:

p. 12, 'After 5 min of incubation, we monitored the changes in ThT fluorescence as a measure of fibril destabilization and dissociation'; the data are difficult to interpret in light of potential quenching effects of SDS on ThT fluorescence. This question could be addressed by performing fluorescence measurements immediately after adding the SDS, or by using a different method to monitor aSyn fibril levels such as ultracentrifugation coupled with Western blot analysis.

While we appreciate this concern, we do not think that additional experiments would be
necessary as any potential quenching effects of SDS on ThT fluorescence would be the same for all samples.

This statement is unconvincing because SDS could have have differential effects on ThT binding to various fibrillar polymorphs at concentrations of the detergent below those required to induce fibril dissociation. In addition, the extent of fibril dissociation in SDS solutions after only 5 minutes of incubation at 37 deg C is uncertain. Given the limitations of quantifying aSyn fibrils solely via ThT fluorescence measurements, the results would be strengthened if complemented by a different method to monitor aSyn fibril levels, such as ultracentrifugation coupled with Western blot analysis.

We have now conducted additional experiments as shown in new Figure S7, which reproduce our findings. We added a section in material and methods and the following sentence to the result section:

Page 13*: “We obtained similar results when we monitored the SDS-mediated destabilization of the fibrils by centrifugation (Fig. S7).”*
